# An experience with ventriculoperitoneal shunting at keen’s point for hydrocephalus

**DOI:** 10.12669/pjms.343.14081

**Published:** 2018

**Authors:** Muhammad Junaid, Mamoon Ahmed, Mamoon Ur Rashid

**Affiliations:** 1 Dr. Muhammad Junaid, FCPS. Classified Neurosurgeon, PNS Shifa, Karachi, Pakistan; 2 Dr. Mamoon Ahmed, MBBS. Department of Cardiac Surgery, Jinnah Hospital, Lahore, Pakistan; 3 Dr. Mamoon Ur Rashid, MBBS. Khyber Teaching Hospital, Peshawar, Pakistan

**Keywords:** Hydrocephalus, Aqueductalstenosis, Ventriculoperitoneal shunt

## Abstract

**Objective:**

This study was conducted to assess outcomes in patients with hydrocephalus who underwent ventriculoperitoneal shunting at Keen’s point.

**Methods:**

This retrospective study was conducted in Combined Military Hospital (CMH) Peshawar. Time frame was four years from January 2011 to January 2015. The presenting complaints, clinical findings, investigations, treatment plans and surgical outcomes were noted. Ventriculo-Peritoneal (VP) shunting was done at Keen’s point. The presence of shunt complications in the first week post-surgery was noted and at a three-month follow up in the outpatient department. General condition of the patient, shunt complications, presence of seizure and worsening of vision were noted.

**Results:**

Study included 143 patients, out of whom 46 were females and 95 were male patients. Most common causes of hydrocephalus were congenital (79). Majority of adults had hydrocephalus due to central nervous system tumors while congenital hydrocephalus in children was most frequently due to aqueductal stenosis. Good clinical improvement was seen in 114 patients after shunt placement, satisfactory in 20 patients, 7 patients died while we observed no change in two patients.

**Conclusion:**

Our experience with VP shunting at Keen’s point resulted in excellent outcomes. It can be used for the management of hydrocephalus both in pediatric as well as adult population.

## INTRODUCTION

Hydrocephalus is defined as a condition in which there is abnormal accumulation of cerebrospinal fluid in the ventricles of the brain. It is mostly associated with an increase in the Intracranial Pressure (ICP).

The main surgical strategy to manage this problem is the placement of Ventriculoperitoneal (VP) shunt. VP shunt insertion is the most common procedure done in neurosurgery and also remains the gold standard surgical procedure to manage Hydrocephalus (HCP).^1^,^2^ More than 40,000 of these procedures are performed annually in the USA, costing over $ 1 billion.^3^,^4^

VP shunt is a CSF diversion device, basically a tube, having a pressure-regulating valve that carries CSF from the ventricular system to another absorptive surface outside the brain such as the peritoneum. However it has very high failure rate, which is almost 40% to 50% for pediatric patients and 30% for adults.^5^,^6^ The failures are mostly related to the occlusion of proximal end of catheters.^7^ The alternate sites for placement of shunt include the right atrium (ventriculoatrial shunt), pleural cavity (ventriculo-pleural shunt), and gallbladder.^8^

Complications of shunts include shunt malfunction, shunt failure, and shunt infection along with infection of shunt tract. Malfunction is the most frequent cause of shunt revision.^9^,^10^

VP shunting involves identification of external landmarks to help in determining the shunt’s entry point. Different locations are used for placing the catheter of the shunt. These may be occipital (Frazier’s point or Dandy’s point), parietal (Keen’s point) or the frontal (Kocher’s point). Keen’s point at the parietal side is almost 2.5-3 cm posterior and superior to the pinna while the direction and length of shunt insertion is almost 4-5 cm perpendicular to the cortex. Insertion of VP shunt is preferred at parietooccipital area because the frontal lobe has much lower seizure threshold than the occipital or parietal lobe and hence may lead to post-operative seizures. The distance between parietooccipital area and peritoneal cavity is also shorter which avoids the need of an additional incision.^11^

We tried to evaluate the success of VP shunting at Keen’s point in our peripheral neurosurgical setups. The result may help in defining the standard protocols for managing hydrocephalus in developing and underdeveloped countries.

## METHODS

A retrospective study of all cases operated at our center over a period of four years from January 2011 to January 2015 was carried out. The study took place at Combined Military Hospitals at Quetta and Peshawar, from 2011-2012 and 2012-2015 respectively. All the patients, who presented, were investigated and operated for hydrocephalus with primary VP shunting were enrolled in this study. Patients were not considered for an Endoscopic Third Ventriculostomy (ETV) because of non-availability of this facility. An informed consent was obtained from all the patients or their guardians and demographic data was noted. The presenting complaints, clinical findings, details of investigations, details of treatment, along with the surgical outcome of patients were noted. All the data was collected in Microsoft Excel and later analyzed using SPSS 21 software.

Base line investigations were carried out for every patient, which included Full Blood Count (FBC), Liver Function Tests (LFTs), Renal Function Tests (RFTs), screening for hepatitis B, hepatitis C and HIV and serum electrolytes. MRI brain was carried for diagnosis of hydrocephalus. Pre-operative antibiotic prophylaxis was done with 2^nd^ generation cephalosporins. Three doses of antibiotics were given.

All the patients had the VP shunt inserted at Keen’s point. Parieto-occipital area was shaved and the skin was cleaned from head to below the umbilicus. Drapes were applied, while Keen’s point as well as an abdominal site for peritoneal access was marked with a sterile needle. The patient was placed in supine position with the head turned 90 degrees to the opposite side of shunt placement. We used medium gradient Chhabra shunt in all patients below 15 years of age. This shunt was preferred relative to other available shunts because of unique spring system of the shunt due to which it is housed in the subcutaneous tissue of the neck. The skin in the infants and children is fragile and necrosis can occur with other shunts but since Chhabra shunt is mainly retained in the subcutaneous tissue of the upper neck, it rarely causes necrosis of the skin. These findings were observed by the operating surgeon. After verifying the shunt system for patency, a small semilunar scalp flap was raised at Keen’s point in a way that the point was at the center of the flap. A small retractor was placed in the incision in order to expose the skull while a small burr hole was made with a scalpel to expose the dura.

A small incision was made in abdomen, almost 2 cm superior and lateral to the umbilicus and carried down to the level of anterior layer of rectus sheath. A shunt passer was then tunneled subcutaneously from the abdominal incision to head incision. Distal aspect of shunt system was then passed through the shunt passer, avoiding its contact with the exposed skin. The shunt passer was later on withdrawn from the patient and after incising the dura with a size 11 blade, the ventricular catheter was introduced into the ventricles and connected to the rest of the shunt system using the connector. The ventricular catheter was then sutured to the pericranium with silk 3/0.

Moving back to the abdominal incision, the anterior layer of the rectus sheath was incised, as the muscle was dissected and peritoneum opened. The abdominal catheter was inserted into peritoneal cavity and cavity was closed by vicryl 3/0. The abdominal skin and scalp were closed with nylon 3/0.

The presence of shunt complications in the first week post-surgery was noted. The clinical outcome of each patient was assessed at a three-month follow up in the outpatient department. General condition of patient, vomiting, bulging of fontanella, shunt complications, presence of seizures, fronto-occipito cranial diameter, excessive crying of child, feeding status were the parameters noted on the follow-up in children.

## RESULTS

We included a total of 143 patients, out of whom 46 were females while 95 were male patients. There were no drop outs from the study. The mean age was 21.56 years with a standard deviation of 23.50 years.

Right sided VP shunting was performed in 114 (78.6%) patients while 31 (21.4%) had left sided shunting. Recommended site is right sided approach but in some of the patients due to presence of tumors, adhesions and injuries from previous trauma on right side, left sided approach was done. The causes of Increased Intracranial Pressure in our subjects (N=145) are shown in Table-I..

**Table-I:** Causes of increased Intracranial Pressure in our subjects (N=145)

Causes of hydrocephalus	Frequency	Percentage
**Congenital causes**	**79**	**54.5**
a.Aqueductal stenosis	32	22
b.Communicating hydrocephalus	17	11.7
c.Dandy-Walker malformation	4	2.7
d.Congenital infections	5	3.4
e.Others	21	14.5
**Acquired causes**	**66**	**45.5**
f.CNS tumours	25	17.2
g.CNS Infections	9	6.2
h.Traumatic Hemorrhage	5	3.4
i.Traumatic brain injury	8	5.5
j.Post tuberculous meningitis	8	5.5
k.Post-pyogenic meningitis	6	4.1
l.Idiopathic	5	3.4

Vomiting, bulging of fontanelle, frontooccipitocranial diameter, excessive crying of child, feeding status were the parameters noted on the follow-up in children. In three months follow up, good clinical improvement was seen in 114 (78.6%) patients, satisfactory in 20 patients while no change was observed in based on the above parameters. Seven (2.5%) patients died while we observed no change in two (1.9%) patients. Various complications noted are given in Table-II. Shunt malfunction was the most common complication. It included both partial and complete blockage of the ventricular and/or peritoneal catheter. The patients were re-operated and shunt procedure was repeated.

**Table-II:** Complications of ventriculo-peritoneal shunt procedure.

Complications	Frequency	Percentage
Shunt malfunction	26	18
Intracranial migration of shunt	1	0.6
Infections	3	2
Seizures	2	1.3

One of the complications noted was the intracranial migration of ventricular catheter in a child of three years of age. This patient also developed meningitis. Patient was re-operated and catheter was removed. All cases of shunt malfunctions were found in infants of less than 10 months of age. Seizures were noted in two infants of ages nine months and one year. Infections were noted in three patients of ages one year, 16 months and 18 months.

## DISCUSSION

Our study was carried out at general neurosurgical unit of tertiary care facility of Khyber Pakhtunkhwa province of Pakistan. As there are only a few units for neurosurgery in the province, hence we had patients from all areas of the provinces.

The number of male patients in our study was more compared to female patients, which is consistent with other studies carried out around the globe.^12^ Majority of children were below 18 years of age and all the patients below eight years reported with congenital hydrocephalus. Although post infectious hydrocephalus is also common amongst the children in low income countries,^13^ but we didn’t see any such patient. Aqueductal stenosis 20.77% was common causes of congenital hydrocephalus in our study.

We achieved good results in 79.47% of the patients. All the patients, in whom no change was observed even after treatment, had post-traumatic communicating hydrocephalus. Patients who are managed with VP shunts must be periodically evaluated to check for any complication. The complication rate of the VP shunt surgery ranges around 30% to 40%, with some studies having failure rate up to 46%^14^, with shunt infection and malfunction of ventricular catheter being the most frequent types of major complications.^5^ In our study most frequent complication noted was shunt malfunction 16.23%. Shunt malfunction was initially assessed by pressing on the reservoir and seeing if it empties into the distal catheter which was considered suggestive of patency. Refilling of the reservoir was considered suggestive of a patent proximal catheter. In doubtful cases, the shunt was tapped and a repeat CT scan was done to assess the degree of hydrocephalus. Blind insertion of the catheter using an external landmark, like Keen’s point, as a reference can be associated with suboptimal positioning of the ventricular catheter along with an increased risk of complications. A study carried out in Japan showed the inappropriate positing to be around 29.7%, while none of our patient had to undergo a repeat surgery for this problem.^15^

Headache is the common presenting symptom, while visual deterioration is the most serious consequence of increased intracranial pressure. Patients undergo CSF diversion procedures like VP shunting to lower the increased intracranial pressure and protect vision.^16^,^17^ The most common presenting complaints in our patients having normal pressure hydrocephalus were gait ataxia and sphincter disturbance. Majority of the children presented with macrocephaly while three presented with papilloedema and sixth nerve palsy.

Previous studies state hemorrhage to have the most detrimental effect on the shunt functioning.^18^,^19^ Shunt may block due to clogging of red blood cells and platelet micro thrombi due to intra-cerebral or intra-parenchymal hemorrhage.^20^ Although brain tumor and normal pressure hydrocephalus are also documented to be the causes of short time malfunctioning of VP shunt, but we did not see any major complication in our patients suffering from these problems too.

Development of hydrocephalus following cranial surgery may be attributed to the damage that occurred to cells of the choroid plexus and other nearby tissues during the surgical procedure^18^ Similarly, the injury to tissues and extensive manipulation that is done during resection of neoplasm, along with alterations in the cerebral blood flow and auto-regulation that occur after the procedure, may result in early shunt failure in patients having brain tumors.^21^

The high success rate proves that VP shunting at Keen’s point to treat hydrocephalus, can be carried out at peripheral setups even in developing countries. We recommend this treatment in all setups where the latest equipment is not available.

## CONCLUSION

In our experience, the results of VP shunting at Keen’s point proved to be a successful procedure even at peripheral neurosurgical centers, with the non-availability of ETV. Therefore it can be used for the management of hydrocephalus, both in pediatric as well as adult population. High rates of failure are mentioned in some studies^6^,^7^ but in our institutions results were reasonably good with success rate of 79.37% at three months from procedure day.

### Authors’ Contributions

**MJ:** Contribution to conception, design and acquisition of data.

**MA:** Analysis and interpretation of data, drafting the manuscript.

**MUR:** Conception and design, revision of manuscript
